# Supplementary material for: Placental Changes and Neuropsychological Development in Children—A Systematic Review
Source: Cells. 2023 Jan 28;12(3):435. doi: 10.3390/cells12030435 (PMC9913696; doi:10.3390/cells12030435)
Supplement: Supplementary file 1 [file cells-12-00435-s001.zip › Supplementary Table S1 Search strategy.pdf]

Supplementary Table S1. Search strategy.

Medline (Ovid MEDLINE(R) and Epub Ahead of Print, In-Process, In-Data-Review & Other Non-Indexed Citations, Daily and Versions <1946 to May 11, 2022>)

Last run May 13, 2022

Search terms			Number of hits
Placenta			
	1	exp Placenta/ or exp Placenta Diseases/ or Pre-Eclampsia/	111,899
	2	(placent* or "pre eclampsia" or preeclampsia or "pregnancy toxem*" or "edema proteinuria hypertension gestosis" or "oedema proteinuria hypertension gestosis" or "toxemia of pregnancy" or "eph complex" or "eph toxemia*" or "eph gestosis" or chorioamnionit* or amnionitis or amnionitides or funisitis or funisitides).ab,kf,ti.	145,250
	3	1 or 2	172,583
Age groups			
	4	exp Child/ or Adolescent/ or Infant/	3,515,980
	5	(babies or baby or boy or boys or child* or girl* or infant* or paediatric* or pediatric* or preschool* or pre-school* or toddler* or kindergar#en* or prekindergar#en* or pre-k or offspring or kid or kids or teen* or youth* or youngster* or adolescent* or adolescence or juvenile* or minors).ab,kf,ti.	2,542,927
	6	4 or 5	4,376,421
Study types			
	7	Cohort Studies/ or Follow-Up Studies/ or Longitudinal Studies/ or Prospective Studies/ or Retrospective Studies/ or Epidemiologic Studies/	2,355,438
	8	(cohort* or incidence or "follow up*" or longitudinal* or prospectiv* or retrospectiv* or "case control*" or "case compar*" or "case-compeer*" or "case referrent" or "case base stud*" or epidemiolog* or (Panel* adj7 stud*) or "odds ratio*" or "relative risk*" or subcohort* or "sub cohort*" or "long term" or longterm or registry or registries or register* or "cross section*" or exposure).ab,kf,ti.	5,837,094
	9	7 or 8	6,389,455
Hypersensitivity			
	10	Hypersensitivity/ or exp Hypersensitivity, Immediate/ or exp Hypersensitivity, Delayed/ or exp Bronchitis/	340,138
	11	(hypersensitiv* or allergy or allergic or allergies or "airway hyper responsive*" or asthma* or bronchitis or bronchiolitis or wheeze).ab,kf,ti.	413,313
	12	(anaphylaxis or anaphylactic or anaphylactoid or "atopic conjunctiviti*" or "vernal conjunctiviti*" or "vernal keratoconjunctiviti*" or "giant papillary conjunctiviti*" or "airway hyper-responsiveness" or "exercise-induced bronchospasm*" or pollinosis or pollinoses or "hay fever" or hayfever or urticaria* or hives or angi#edema* or "quincke's edema*" or "quincke's oedema*" or "quincke edema*" or "quincke oedema*" or "quinckes edema*" or "quinckes oedema*" or "angioneurotic edema*" or "angioneurotic oedema*" or "c1 inhibitor deficienc*" or "c1 esterase inhibitor deficienc*" or urticaria* or "atopic eczema*" or "atopic dermatitis").ab,kf,ti.	85,195
	13	(dermatiti* adj2 (photosensitiv* or photoallerg*)).ab,kf,ti.	366
	14	(eosinophilic adj2 (esophagitides or esophagitis)).ab,kf,ti.	3,024
	15	10 or 11 or 12 or 13 or 14	530,330
Psychiatric / psychological conditions			
	16	Mental Disorders/ or exp Anxiety Disorders/ or exp "Bipolar and Related Disorders "/ or exp "Feeding and Eating Disorders "/ or exp	723,676

		Mood Disorders/ or exp Neurodevelopmental Disorders/ or exp "Schizophrenia Spectrum and Other Psychotic Disorders "/	
	17	((cognitive or neurocognitive) adj3 (impair* or dysfunction* or disorder* or declin*)).ab,kf,ti.	138,335
	18	(intellectual* adj2 (disabilit* or disable*)).ab,kf,ti.	22,898
	19	("mental disorder*" or "psychiatric illness*" or "psychiatric disease*" or "mental illness*" or "mentally ill" or "psychiatric disorder*" or "psychiatric diagnos*" or anxiety* or neurotic or neuroses or neurosis or psychoneuros* or "obsessive compulsive" or "anankastic personalit*" or hoarding or hoarder* or panic* or phobic or phobia* or claustrophobi* or bipolar* or "manic depress*" or "manic disorder*" or anorexia or bulimia or "avoidant restrictive food intake disorder*" or "food neophobia*" or arfid or "binge eating" or diabulim* or "food addict*" or "compulsive eating" or "night eating syndrome" or pica or "rumination syndrome*" or "rumination disorder*" or "mood disorder*" or "affective disorder*" or depression* or depressive or depressed or cyclothymi* or neurodevelopment* or "behavior disorder*" or "child development disorder*" or autism or autist* or asperger* or "communication disorder*" or "motor skills disorder*" or mutism or "reactive attachment disorder" or schizophren* or "stereotypic movement disorder*" or "tic disorder*" or "affective psychotic disorder*" or paranoi* or "mental deterioration" or "internali#ing symptom*" or "externali#ing symptom*" or "internali#ing disorder*" or "negative affect*" or "externali#ing disorder*").ab,kf,ti.	1,051,987
	20	((feeding or eating or appetite) adj2 disorder*).ab,kf,ti.	26,524
	21	(("attention deficit" adj4 disorder*) or "disruptive behavioral disorder*").ab,kf,ti.	31,735
	22	(development* adj1 (disabilit* or disorder* or deviation*)).ab,kf,ti.	18,042
	23	(learning adj1 (disabilit* or disorder*)).ab,kf,ti.	10,146
	24	16 or 17 or 18 or 19 or 20 or 21 or 22 or 23	1,473,385
Cancer			
	25	exp Neoplasms/	3,685,066
	26	(neoplas* or cancer* or tumor* or tumour* or malignan* or lymphoma* or neuroblastoma* or nephroblastoma* or carcinoma*).ab,kf,ti.	3,853,047
	27	25 or 26	4,815,549
All diseases/conditions combined with OR			
	28	15 or 24 or 27	6,686,820
Placenta, study types, age groups and diseases/conditions combined with AND			
	29	3 and 6 and 9 and 28	2,603
Fetal programming			
	30	Prenatal Exposure Delayed Effects/	32,919
	31	("fetal programming*" or "foetal programming*" or "developmental origin? of health and disease*" or "developmental origin? of health and adult disease*" or "Barker?? hypothesis").ab,kf,ti.	3,314
	32	30 or 31	35,308
Fetal programming, placenta and study type combined with AND			
	33	32 and 3 and 9	1,663
Both strategies combined with OR			
	34	29 or 33	3,798
Limits			
	35	(animals/ not humans/) or comment/ or editorial/ or exp review/ or meta analysis/ or consensus/	9,209,370
	36	34 not 35	2,341
	37	limit 36 to english language	2,242
	38	limit 37 to yr="2000 - 2023"	1,968

Embase (Embase.com)

Last run May 13, 2022

Search terms			Number of hits
Placenta			
	1	'placenta'/exp OR 'placenta disorder'/exp OR 'pregnancy toxemia'/de OR 'preeclampsia'/de	167,988
	2	placent*:ti,ab,kw OR 'pre eclampsia':ti,ab,kw OR preeclampsia:ti,ab,kw OR 'pregnancy toxem*':ti,ab,kw OR 'oedema proteinuria hypertension gestosis':ti,ab,kw OR 'edema proteinuria hypertension gestosis':ti,ab,kw OR 'toxemia of pregnancy':ti,ab,kw OR 'eph complex':ti,ab,kw OR 'eph toxemia*':ti,ab,kw OR 'eph gestosis':ti,ab,kw OR chorioamnionit:ti,ab,kw OR amnionitis:ti,ab,kw OR amnionitides:ti,ab,kw OR funisitis:ti,ab,kw OR funisitides:ti,ab,kw	189,117
	3	#1 OR #2	233,150
Age groups			
	4	'child'/de OR 'infant'/de OR 'high risk infant'/de OR 'adolescent'/de OR 'juvenile'/de	3,483,700
	5	babies:ti,ab,kw OR baby:ti,ab,kw OR boy:ti,ab,kw OR boys:ti,ab,kw OR child*:ti,ab,kw OR girl*:ti,ab,kw OR infant*:ti,ab,kw OR paediatric*:ti,ab,kw OR pediatric*:ti,ab,kw OR preschool*:ti,ab,kw OR toddler*:ti,ab,kw OR kindergar*en*:ti,ab,kw OR prekindergar*en*:ti,ab,kw OR 'pre k':ti,ab,kw OR offspring:ti,ab,kw OR kid:ti,ab,kw OR kids:ti,ab,kw OR teen*:ti,ab,kw OR youth*:ti,ab,kw OR youngster*:ti,ab,kw OR adolescent*:ti,ab,kw OR adolescence:ti,ab,kw OR juvenile*:ti,ab,kw OR minors:ti,ab,kw	3,274,351
	6	#4 OR #5	4,689,111
Study types			
	7	'cohort analysis'/de OR 'case control study'/exp OR 'follow up'/de OR 'longitudinal study'/exp OR 'prospective study'/de OR 'retrospective study'/de OR 'epidemiology'/exp	6,864,943
	8	cohort*:ti,ab,kw OR incidence:ti,ab,kw OR 'follow up*':ti,ab,kw OR longitudinal:ti,ab,kw OR prospectiv*:ti,ab,kw OR retrospectiv*:ti,ab,kw OR 'case control*':ti,ab,kw OR 'case compar*':ti,ab,kw OR 'case-compeer*':ti,ab,kw OR 'case referrent':ti,ab,kw OR 'case base stud*':ti,ab,kw OR epidemiolog*:ti,ab,kw OR 'odds ratio*':ti,ab,kw OR 'relative risk*':ti,ab,kw OR subcohort*:ti,ab,kw OR 'sub cohort*':ti,ab,kw OR 'long term':ti,ab,kw OR longterm:ti,ab,kw OR registry:ti,ab,kw OR registries:ti,ab,kw OR register*:ti,ab,kw OR 'cross section*':ti,ab,kw OR exposure:ti,ab,kw OR ((panel* NEAR/7 stud*):ti,ab,kw)	8,253,865
	9	#7 OR #8	10,534,011
Hypersensitivity			
	10	'hypersensitivity'/de OR 'delayed hypersensitivity'/exp OR 'immediate type hypersensitivity'/exp OR 'atopic dermatitis'/de OR 'bronchitis'/exp	593,744
	11	hypersensitiv*:ti,ab,kw OR allergy:ti,ab,kw OR allergic:ti,ab,kw OR allergies:ti,ab,kw OR 'airway hyper responsive*':ti,ab,kw OR asthma*:ti,ab,kw OR bronchitis:ti,ab,kw OR bronchiolitis:ti,ab,kw OR	619,800

		wheeze:ti,ab,kw OR 'atopic eczema*':ti,ab,kw OR 'atopic dermatitis':ti,ab,kw	
	12	anaphylaxis:ti,ab,kw OR anaphylactic:ti,ab,kw OR anaphylactoid:ti,ab,kw OR 'atopic conjunctiviti*':ti,ab,kw OR 'vernal conjunctiviti*':ti,ab,kw OR 'vernal keratoconjunctiviti*':ti,ab,kw OR 'giant papillary conjunctiviti*':ti,ab,kw OR 'airway hyper-responsiveness':ti,ab,kw OR 'exercise-induced bronchospasm*':ti,ab,kw OR pollinosis:ti,ab,kw OR pollinoses:ti,ab,kw OR 'hay fever':ti,ab,kw OR hayfever:ti,ab,kw OR hives:ti,ab,kw OR angi*edema*:ti,ab,kw OR 'quincke* edema*':ti,ab,kw OR 'quincke* oedema*':ti,ab,kw OR 'angioneurotic edema*':ti,ab,kw OR 'angioneurotic oedema*':ti,ab,kw OR 'c1 inhibitor deficienc*':ti,ab,kw OR 'c1 esterase inhibitor deficienc*':ti,ab,kw OR urticaria*:ti,ab,kw	89,657
	13	(dermatiti* NEAR/2 (photosensitiv* OR photoallerg*)):ti,ab,kw	592
	14	(eosinophilic NEAR/2 (esophagitides OR esophagitis)):ti,ab,kw	6,466
	15	#10 OR #11 OR #12 OR #13 OR #14	832,004
Psychiatric / psychological conditions			
	16	'mental disease'/de OR 'developmental disorder'/exp OR 'anxiety disorder'/exp OR 'autism'/exp OR 'attention deficit disorder'/de OR 'eating disorder'/exp OR 'mood disorder'/exp OR 'learning disorder'/exp OR 'communication disorder'/exp OR 'schizophrenia spectrum disorder'/exp	1,407,294
	17	((cognitive OR neurocognitive) NEAR/3 (impair* OR dysfunction* OR disorder* OR declin*)):ti,ab,kw	209,957
	18	(intellectual* NEAR/2 (disabilit* OR disable*)):ti,ab,kw	33,052
	19	'mental disorder*':ti,ab,kw OR 'psychiatric illness*':ti,ab,kw OR 'psychiatric disease*':ti,ab,kw OR 'mental illness*':ti,ab,kw OR 'mentally ill':ti,ab,kw OR 'psychiatric disorder*':ti,ab,kw OR 'psychiatric diagnos*':ti,ab,kw OR anxiety*:ti,ab,kw OR neurotic:ti,ab,kw OR neuroses:ti,ab,kw OR neurosis:ti,ab,kw OR psychoneuros*:ti,ab,kw OR 'obsessive compulsive':ti,ab,kw OR 'anankastic personalit*':ti,ab,kw OR hoarding:ti,ab,kw OR hoarder*:ti,ab,kw OR panic*:ti,ab,kw OR phobic:ti,ab,kw OR phobia*:ti,ab,kw OR claustrophobi*:ti,ab,kw OR bipolar*:ti,ab,kw OR 'manic depress*':ti,ab,kw OR 'manic disorder*':ti,ab,kw OR anorexia:ti,ab,kw OR bulimia:ti,ab,kw OR 'avoidant restrictive food intake disorder*':ti,ab,kw OR 'food neophobia*':ti,ab,kw OR arfid:ti,ab,kw OR 'binge eating':ti,ab,kw OR diabulim*:ti,ab,kw OR 'food addict*':ti,ab,kw OR 'compulsive eating':ti,ab,kw OR 'night eating syndrome':ti,ab,kw OR pica:ti,ab,kw OR 'rumination syndrome*':ti,ab,kw OR 'rumination disorder*':ti,ab,kw OR 'mood disorder*':ti,ab,kw OR 'affective disorder*':ti,ab,kw OR depression*:ti,ab,kw OR depressive:ti,ab,kw OR depressed:ti,ab,kw OR cyclothymi*:ti,ab,kw OR neurodevelopment*:ti,ab,kw OR 'behavior disorder*':ti,ab,kw OR 'child development disorder*':ti,ab,kw OR autism:ti,ab,kw OR autist*:ti,ab,kw OR asperger*:ti,ab,kw OR 'communication disorder*':ti,ab,kw OR 'motor skills disorder*':ti,ab,kw OR mutism:ti,ab,kw OR 'reactive attachment disorder':ti,ab,kw OR schizophren*:ti,ab,kw OR 'stereotypic movement disorder*':ti,ab,kw OR 'tic disorder*':ti,ab,kw	1,409,508

		OR 'affective psychotic disorder*':ti,ab,kw OR paranoi*:ti,ab,kw OR 'mental deterioration':ti,ab,kw OR 'internali*ing symptom*':ti,ab,kw OR 'externali*ing symptom*':ti,ab,kw OR 'internali*ing disorder*':ti,ab,kw OR 'negative affect*':ti,ab,kw OR 'externali*ing disorder*':ti,ab,kw	
	20	((feeding OR eating OR appetite) NEAR/2 disorder*):ti,ab,kw	34,162
	21	(('attention deficit' NEAR/4 disorder*):ti,ab,kw) OR 'disruptive behavioral disorder*':ti,ab,kw	40,760
	22	(development* NEAR/1 (disabilit* OR disorder* OR deviation*)):ti,ab,kw	25,690
	23	(learning NEAR/1 (disabilit* OR disorder*)):ti,ab,kw	14,111
	24	#16 OR #17 OR #18 OR #19 OR #20 OR #21 OR #22 OR #23	2,139,096
Cancer			
	25	'neoplasm'/exp	5,622,017
	26	neoplas*:ti,ab,kw OR cancer*:ti,ab,kw OR tumor*:ti,ab,kw OR tumour*:ti,ab,kw OR malignan*:ti,ab,kw OR lymphoma*:ti,ab,kw OR neuroblastoma*:ti,ab,kw OR nephroblastoma*:ti,ab,kw OR carcinoma*:ti,ab,kw	5,251,175
	27	#25 OR #26	6,661,705
All diseases/conditions combined with OR			
	28	#15 OR #24 OR #27	9,332,358
Placenta, study types, age groups and diseases/conditions combined with AND			
	29	#3 AND #6 AND #9 AND #28	5,247
Fetal programming			
	30	'fetal programming*':ti,ab,kw OR 'foetal programming*':ti,ab,kw OR 'prenatal programming*':ti,ab,kw OR 'developmental origin* of health and disease*':ti,ab,kw OR 'developmental origin* of health and adult disease*':ti,ab,kw OR 'barker* hypothesis':ti,ab,kw	5,192
Fetal programming, placenta and study type combined with AND			
	31	#3 AND #9 AND #30	661
Both strategies combined with OR			
	32	#29 OR #31	5,774
Limits			
	33	#32 NOT ('conference abstract'/it OR 'conference review'/it OR 'editorial'/it OR 'letter'/it OR 'note'/it OR 'review'/it)	3,076
	34	#33 NOT ([animals]/lim NOT [humans]/lim) AND [english]/lim AND [2000-2022]/py	2,419
